# LncTUG1 ameliorates renal tubular fibrosis in experimental diabetic nephropathy through the miR-145-5p/dual-specificity phosphatase 6 axis

**DOI:** 10.1080/0886022X.2023.2173950

**Published:** 2023-02-16

**Authors:** Taoxia Wang, Shubei Cui, Xiaoli Liu, Li Han, Xiaoting Duan, Shuning Feng, Sen Zhang, Guiying Li

**Affiliations:** a Department of Nephrology, Affiliated Hospital of Hebei University of Engineering, Hebei, China; b The First Department of Orthopedics, Handan Central Hospital, Handan, China; c State Key Laboratory of Bioactive Substances and Functions of Natural Medicines, Institute of Materia Medica, Chinese Academy of Medical Sciences & Peking Union Medical College, Beijing, P. R. China

**Keywords:** Diabetic nephropathy, interstitial fibrosis, lncTUG1, miR-145-5p, DUSP6, inflammation

## Abstract

The renal interstitial fibrosis contributes to the progression and deterioration of diabetic nephropathy (DN). Long noncoding RNA taurine-up-regulated gene 1 (TUG1) in kidneys may be down-regulated by hyperglycemia. We aim to explore its role in tubular fibrosis caused by high glucose and the possible target genes of TUG1. In this study, a streptozocin-induced accelerated DN mouse model and a high glucose-stimulated HK-2 cells model was established to evaluate TUG1 expression. Potential targets of TUG1 were analyzed by online tools and confirmed by luciferase assay. A rescue experiment and gene silencing assay were used to investigate whether TUG1 plays its regulation role *via* miR-145-5p/dual-specificity phosphatase 6 (DUSP6) in HK2 cells. The effects of TUG1 on inflammation and fibrosis in high glucose treated tubular cells were evaluated by *in vitro* study, as well as *in vivo* DN mice model through AAV-TUG1 delivery. Results showed TUG1was downregulated in HK2 cells incubated with high glucose while miR-145-5p was upregulated. Overexpression of TUG1 alleviated renal injury by suppressing inflammation and fibrosis *in vivo*. Overexpression of TUG1 inhibited HK-2 cell fibrosis and relieved the inflammation. A mechanism study demonstrated that TUG1 directly sponged to miR-145-5p, and DUSP6 was identified as a target downstream of miR-145-5p. In addition, miR-145-5 overexpression and DUSP6 inhibition countervailed the impacts of TUG1. Our findings revealed that TUG1 overexpression alleviates kidney injury in DN mice and decreases the inflammatory response and fibrosis of high glucose-stimulated HK-2 cells *via* miR-145-5p/DUSP6 axis.

## 1. Introduction

As one of the most common chronic diseases, knowledge of diabetes has been quickly enriched in decades. The occurrence and development of diabetes are the results of the interaction of genes and the environment. Hyperglycemia generally affects several important organs, including kidney [1]. The etiology and pathogenesis of diabetic nephropathy (DN) are complicated and have not been fully elucidated. If DN cannot be controlled effectively, most DN patients will progress in to end-stage renal disease (ESRD), whose typical pathological characteristics are interstitial fibrosis and glomerulosclerosis [2]. Many factors contribute to the DN caused kidney fibrosis, such as urinary protein, high glucose, lipidemia, hypoxia, inflammation, and terminal glycosylation products [3]. The researchers indicate that hyperglycemia leads to the activation of tissue angiotensin II [4], and angiotensin II stimulates the synthesis of extracellular matrix (ECM) proteins and reduces the activity of proteolytic enzymes. Deposition of ECM proteins is the direct cause of fibrosis. The accumulation of extracellular matrix leads to the glomerular endothelial cell hypertrophy and apoptosis of renal tubular cells, and then glomerulosclerosis and renal tubulointerstitial fibrosis [5,6].

The TGF-β signaling pathway is involved in the occurrence and development of renal fibrosis [6–8]. The progression of diabetic nephropathy (DN) is also affected by a combination of genetic and environmental factors [1], including epigenetic modifications by lncRNAs or miRNAs [9]. Both podocyte and tubular cell injuries are important events in the initiation of diabetic nephropathy, but fibrosis in the interstitial tubular area is dominant in whole kidney pathological events [10,11]. Recent studies indicate that miRNAs are also involved in the process of tubular injury and promote or mediate the process of renal tubular fibrosis [12]. In addition to the classical TGF-β/Smads signaling pathway, TGF-β is also involved in a variety of cellular signal pathways to regulate the occurrence and development of fibrosis, such as mitogen-activated protein kinase (MAPK) and Wnt/β - Catenin, mTOR pathway, etc. [13]. The activation of the extracellular signal-regulated kinases 1 and 2 (ERK1/2) MAPK signal pathway has been reported to be involved in TGF-β induced kidney fibrosis, through direct or indirect processes, TGF-β activates ERK1/2, which further phosphorylates downstream target proteins and promotes the process of cell fibrosis [14].

Dual-specificity phosphatases (DUSPs) are negative regulators of MAPK signal transduction and can dephosphorylate p38, c-Jun N-terminal kinase (JNK) and ERK under different conditions [15]. EERK1/2 are important extracellular signals transductor. DUSP6 can specifically dephosphorylate ERK1/2 on tyrosine and serine/threonine residues, resulting in the inactivation of ERK1/2 *in vitro* and *in vivo* [16]. A previous study indicated that DUSP6 expression levels were significantly decreased in kidney tissues of DN mice, as well as in high glucose (HG)‑treated podocyte cells [17]. DUSP6 overexpression enhanced podocyte cell viability and increased integrity biomarkers (synaptopodin and nephrin), and also reduced the levels of reactive oxygen species (ROS) and inflammatory cytokines, including interleukin (IL)‑1β, IL‑ 6, and tumor necrosis factor‑α (TNFα) secreted by podocyte cells under HG conditions [17]. However, its role in tubular cells is rarely reported, therefore, in the current study, we are going to investigate whether DUSP6 expression in tubular cells changed under HG stimulation, and further clarify its molecular role in the tubular fibrosis.

Long noncoding RNA (LncRNA) is a class of non-coding RNA with nucleotides longer than 200 bp, and they were once considered not to have important biological functions [18,19]. With the in-depth study of genomics, researchers have found that LncRNAs have multiple important biofunctions, and most are associated with regulation of gene expression [20]. The taurine-up-regulated gene 1 (TUG1) is a 7.1 KB LncRNA, which was initially found to be expressed during retinal development *in vitro* [21]. Previous study suggests that expression of TUG1 may be regulated by hyperglycemia, and its downregulation could lead to the impairment of β-cell function in diabetic mice [22]. The main purpose of this study is to investigate whether TUG1 has a protective effect on HG-induced renal tubular fibrosis and its related molecular mechanism.

## 2. Materials and methods

### 2.1. Cell culture and transfection

Human renal tubular epithelial cell line HK2 was obtained from the cell bank of the Chinese Academy of Sciences (Shanghai, China). Cells were cultured in Roswell Park Memorial Institute-1640 (RPMI-1640) medium supplemented with 10% fetal bovine serum (Solarbio, Beijing, CHN), 100 U/ml penicillin (Solarbio), and 100 μg/ml streptomycin (Solarbio). To evaluate the effect of high glucose on HK2 cells, cells were cultured in a medium containing normal glucose (NG, 5.5 mmol/L) and high glucose (HG, 25 mmol/L) for 72 h.

### 2.2. RNA extraction and quantitative real-time PCR

Cells were lysed by TRIzol reagent (Thermo Fisher Scientific). Total RNA was extracted using RNA Extraction Kit (Solarbio) following the manufacturer’s protocol. After checking the purity and integrity of RNA, a total of 1 μg RNA was reverse transcribed using the TaKaRa One Step RNA PCR Kit (RR024B, TaKaRa, Japan) according to the manufacturer’s instructions by 7500 Real-Time PCR Systems (Applied Biosystems, Foster City, CA). U6 and β-actin served as control. The mRNA levels of TUG1, DUSP6, TNFα, IL-6, fibronectin (FN1) and collagen IV (COL4A2) were determined by quantitative real-time PCR. The data obtained after three independent experiments were calculated by the formula relative quantification (RQ)=2^- ΔΔ CT^ method. All the primers were listed in Table 1.

**Table 1.** Sequences of qRT-PCR primer.

Gene	Sequence
miR-145-5p (human)	F: 5′-CAGTCTTGTCCAGTTTTCCCAG-3′
	R: 5′-TATGCTTGTTCTCGTCTCTGTGTC-3′
U6 (Human)	F: 5′-CTCGCTTCGGCAGCACA −3′
	R: 5′-AACGCTTCACGAATTTGCGT −3′
TUG1 (human)	F: 5′-CAAGAAACAGCAACACCAGAAG-3′
	R: 5′-TAAGGTCCCCATTCAAGTCAGT-3′
Fibronectin (FN1)	F: 5′-ACAACACCGAGGTGACTGAGAC-3′
	R: 5′-GGACACAACGATGCTTCCTGAG-3′
Collagen IV (COL4A2, Human)	F: 5′-GGATAACAGGCGTGACTGGAGT-3′
	R: 5′-CTTTGCCACCAGGCAGTCCAAT-3′
DUSP6 (human)	F: 5′-CTCGGATCACTGGAGCCAAAAC-3′
	R: 5′-CTTTGCCACCAGGCAGTCCAAT-3′
TNFα (human)	F: 5′-CTCTTCTGCCTGCTGCACTTTG-3′
	R: 5′-ATGGGCTACAGGCTTGTCACTC-3′
IL6 (human)	F: 5′-AGACAGCCACTCACCTCTTCAG-3′
	R: 5′-TTCTGCCAGTGCCTCTTTGCTG-3′
β-actin	F: 5′-GTCACAGTGACTGAGCGGCTAA −3′
	R: 5′-CAACTAAGTCTAGTCCGCCTAGA −3′

### 2.3. Cell transfection

The inhibitors of miR-145-5p (Product No.: miR20000437-1-5, miR2N0000002-1-5), negative control inhibitor (miR-145-5p NC), siRNA of DUSP6 (Product No.: siB118593830-1-5, siG000001848A-1-5, and siN0000001-1-5), and siRNA-negative control (siRNA-NC) were purchased from RiboBio (Guangzhou, China). PCDNA3.1-TUG1 and PCDNA3.1 vector were purchased from Sino biological Ltd (Beijing, China). The transfection was performed using Lipofectamine 2000 reagent (Thermo Fisher Scientific) according to the manufacturer’s instructions. Briefly, when the fusion of HK2 cells reach 50–80%, the PCDNA3.1-TUG1, miR-145-5p inhibitor and si-DUSP6, and their corresponding negative control oligonucleotides were diluted in Lipofectamine 2000 agent (11668019, Invitrogen, CA, USA) for transfection. The cells were cultured in a medium without fetal bovine serum at 37 °C in a 5% CO_2_ incubator for 6 h. The medium was then replaced by a complete medium and cultured for another 24 h. The cells transfected with single miR-145-5p inhibitor, miR-145-5p inhibitor plus si-DUSP6, and negative controls were collected for analysis. HK2 cells transfected with PCDNA3.1-TUG1 were screened for stable over-expression colonies, and maintained by appropriate G418 antibiotics.

### 2.4. Luciferase reporter assay

The wild-type (WT) or mutated (Mut) sequence of TUG1 3’UTR and DUSP6 3’UTR were designed and synthesized by RiboBio (Guangzhou, China) and inserted into a pmirGLO Dual-luciferase Vector (Promega, USA). Firstly, the interaction between TUG1 and miR-145-50 was investigated. Briefly, the HK-2 cells were seeded in 24-well plates and co-transfected with miR-145-5p mimics with TUG1-WT or TUG1-Mut using Lipofectamine 2000 (Invitrogen), and miR-NC oligonucleotides were also transfected as negative control. The luciferase activity was then determined after 48 h using the Dual-Luciferase Reporter System (Promega, USA). After that, the interaction between DUSP6 3’UTR with miR-145-5p mimics or miR-NC were analyzed by same procedure.

### 2.5. Cell viability measurement

CCK-8 (Beyotime, Wuhan, China) assay was used to measure cell viability according to the manufacturer’s protocols. The HK2 cells in the logarithmic growth stage were seeded in 96 well plates after transfection, with each well containing 5 × 10^4^ cells. Cells were treated with high (25 mmol/L) or normal (5.5 mmol/L) glucose concentrations for 72 h. The cell viability was measure at 24, 48 and 72h time points respectively. Absorbance value (OD value) at 450 nm wavelength was measured after the CCK-8 reagent was added and incubated at 37°Cfor four hours.

### 2.6. Western blot

The total protein of cells was extracted by RIPA buffer (Solarbio) and were electrophoresed into 8% SDS-PAGE gels and blotted onto PVDF membrane (Millipore, CA, USA). The membrane was blocked by 5% skimmed milk (BD, CA,USA) for 1h. Primary antibodies for TNFα (1:1000, ab92324, Abcam, Cambridge, MA, USA), IL-6 (1:1000, ab259341, Abcam) DUSP6 (1:2000, # 10433-1-AP, Proteintech Group, Inc., USA), Collagen IV (1:5000, #15191-1-AP, Proteintech Group, Inc.), fibronectin (1:1000, #15613-1-AP, Proteintech Group, Inc.) were added and incubated overnight. A secondary antibody (1:5000, # SA00001-2, Proteintech Group, Inc.) was used to incubate for 2 h at room temperature. The bands were examined through an ECL reagent (Beyotime) and analyzed via the Image Lab Software (NIH, USA). The relative expression of the target protein = the gray value of the target band/the gray value of the reference band of β-actin.

### 2.7. Diabetic nephropathy mice model

All experimental protocol with mice was approved by the Ethical Committee of Affiliated Hospital of the Hebei University of Engineering and was performed following the National Institutes of Health Guide for the Care and Use of Laboratory Animals (Approval number was HJ20200605). Male C57BL/6J mice, 6–8 weeks, 18–20 g, were purchased from Vital River (Beijing, China), and underwent uninephrectomy (Unx) to hasten the development of DN, as described previously [11]. Unx mice were maintained in a 12-h light and dark cycle. Diabetic mice were induced by the intraperitoneal repeated injection of a low dose of streptozotocin (STZ) for 5 days with a dose of 55 mg/kg STZ citrate buffer. DN mice were used to induce fasting and water deprivation 12 h before the injection. STZ was injected once a day for 5 consecutive days. One week after the last STZ injection, mice with non-fasting blood glucose ≥15mmol/L were selected for further analysis. Urinary protein could be induced in mice for about 2 weeks after injection. The four urinary mice were sacrificed and kidneys of them were collected for gene expression study. The remaining mice with urinary protein were randomly divided into two groups for the follow-up treatment study: DN-AAV (vector control) group and DN-TUG1 group (*N* = 8). Adeno-associated viruses (AAVs; serotype: 9) containing TUG1 were injected into mice by tail vein, as well as vector control (*N* = 8). All the DN mice received AAV-TUG1 treatment every week. Another eight normal mice without STZ injection were involved as normal control. All the mice were sacrificed after eight weeks since the first AAV injection. During the study period, the mice were raised to the ambient temperature of 18–22 °C, the relative humidity was 50–60%, and the mice were illuminated for 10-14 h a day.

### 2.8. Kidney function measurement

Blood urea nitrogen (BUN) and serum creatinine (Scr) is renal function indicators and their levels in the serum were detected using a Urea Nitrogen Colorimetric Detection Kit (Cat. # EIAGSHC; Invitrogen, USA) and a Creatinine Urinary Detection Kit (cat. # EIACUN; Invitrogen, USA). The urine from each mouse was also collected by metabolic cages, and 24h urinary albumin content for each mouse was measured by mouse albumin ELISA kit (Abcam, USA).

### 2.9. Pathological evaluation and immunohistochemistry

After sacrifice, the kidneys of mice in each group were collected for further evaluation. Renal tissues were fixed with 4% paraformaldehyde, embedded in paraffin. The sections were stained with hematoxylin-eosin (HE) and Masson’s trichrome. The pathological changes in renal tissue were observed under a light microscope. 10 visual fields from each section were taken pictures, and the tubular injury was evaluated by Image-Pro plus 6.0 software (Media Cybernetics, CA, USA). The renal tubular injury was evaluated as: no injury (0 point), injury area <10% (1 point), 10% ≤injury area <25% (2 points), 25% ≤injury area <50% (3 points), 50% ≤ injury area <75% (4 points), injury area ≥75% (5 points).

Aims to evaluate the renal fibrosis, immunohistochemistry of TGFβ1, collagens type I and III was performed with the kidney sections according to the previous studies [23,24]. Antigen retrieval was performed with citrate buffer in a microwave oven. The sections were incubated with primary TGFβ1, collagens type I & III antibodies (Abcam, Cambridge, MA, USA) overnight at 4 °C. The staining was detected with biotin-conjugated goat anti-rabbit IgG (ZSGB-Bio, Beijing, China) and avidin-biotin peroxidase complex ((ZSGB-Bio, Beijing, China). Positive areas were measured using the software Image Pro plus 5.0 (Media Cybernetics, Inc., Rockville, MD, USA) and expressed as percent of positive area per high power field (HPF). Thirty random cortex 400× HPFs were selected and taken pictures under ZEISS Observer A1 microscope (ZEISS, Oberkochen, Germany) for analysis.

### 2.10. Human microarray data collection

The microarray data used in this study were retrieved from the GEO database (http://www.ncbi.nlm.nih.gov/gds/). The following strategy was used: (Homo sapiens) and (Expression profiling by array) and (diabetic nephropathy). We selected data according to the following criteria: each dataset included DN kidney tissue and healthy donor kidney tissues, and each group contained more than four samples. GSE51647 data set (kidney samples from four healthy control and six DN patients) and GSE114477 (kidney samples from four healthy control and four DN patients) were analyzed. No ethical approval or informed consent was required in this study due to the public availability of data in the GEO databases.

GEO2R (http://www.ncbi.nlm.nih.gov/geo/geo2r/) is an online tool provided by GEO. GEO2R is based on the R language limma package (v3.26.8). GEO2R was used to screen differentially expressed lncRNAs, miRNAs and mRNAs

### 2.11. Statistical analysis

The data were statistically processed with GraphPad Prism 8.3 software (GraphPad Software, Inc, La Jolla, CA, USA). The measurement data conforming to the normal distribution were expressed as mean ± SD. The data not conforming to the normal distribution were compared by a nonparametric test. The comparison between multiple groups was analyzed by one-way ANOVA, the Bonferroni *post hoc* test was adopted for comparison between groups. *p* < 0.05 was considered statistically significant.

## 3. Results

### 3.1. Expression of TUG1 and DUSP6 decreases in high glucose treated HK2 cells and kidney tissues in diabetic mice

**Figure 1.**
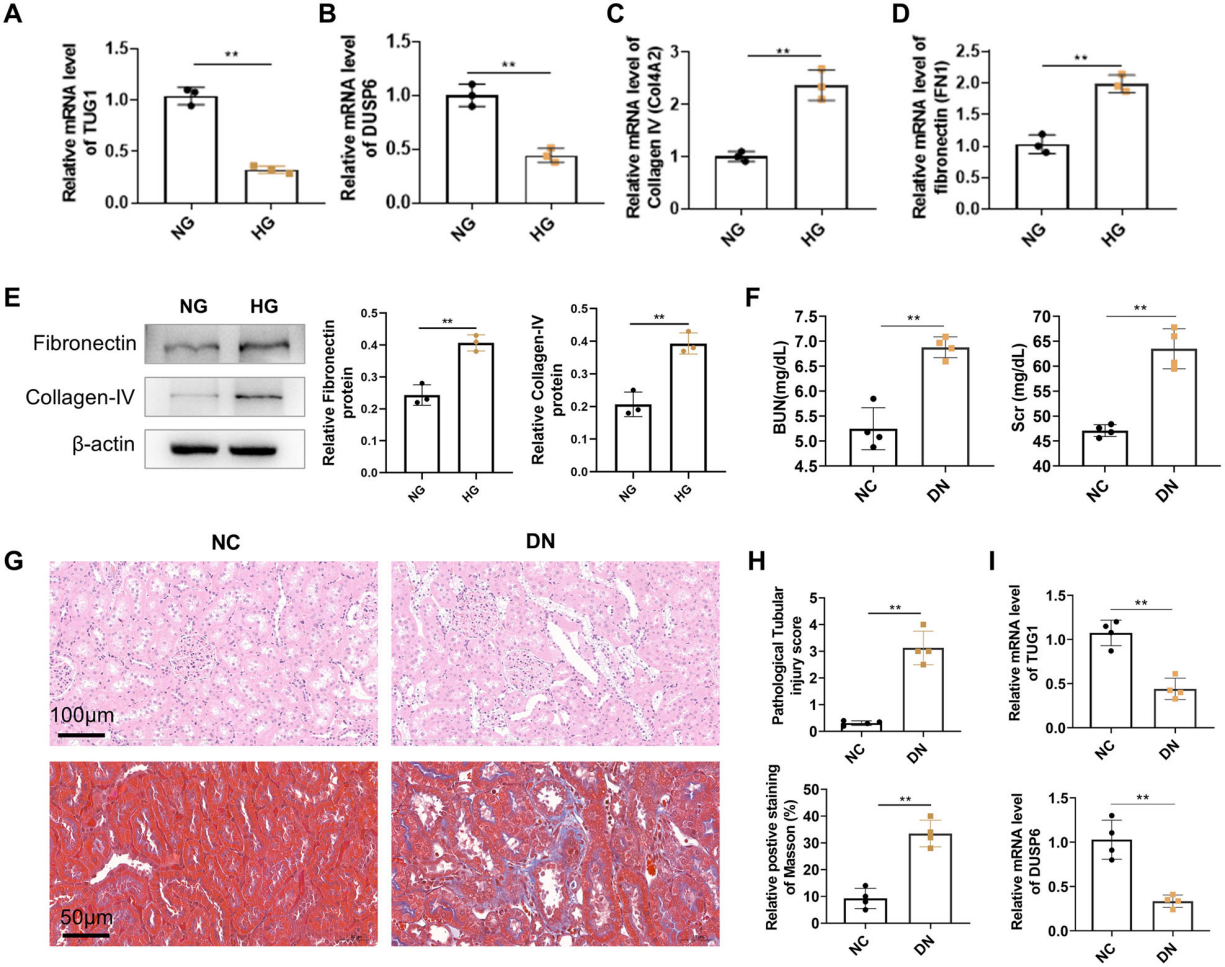
mRNA expressions or protein levels of TUG1, DUSP6, and fibrosis-related genes in HK2 cells treated by normal glucose (NG) and high glucose (HG), and kidney tissues from experimental diabetic nephropathy mice. (A) TUG1 mRNA; (B) DUSP6 mRNA; (C) collagen IV (COL4A2) mRNA; (D) fibronectin (FN1) mRNA. (E) protein levels of fibronectin and collagen IV; (F) BUN and Scr in DN mice. (G) Tubular injury and fibrosis by HE and Masson’s trichrome in DN mice; (H) quantitative analysis of tubular injury and fibrosis; (I) mRNA expression of TUG1 and DUSP6 in kidney tissues from DN and normal control mice. **p < 0.01.

The TUG1 expression was significantly downregulated in HK2 cells treated with high glucose compared with cells treated with normal glucose (Figure 1(A)). The expression of DUSP6 also decreased in high glucose (Figure 1(B)). Further analysis showed that mRNA expression levels of collagen IV (COL4A2) and fibronectin (FN1), important markers of fibrosis, increased in HG-treated cells compared with normal glucose (Figure 1(C,D)). Meanwhile, by western blot, we also confirmed that high glucose induced the higher protein production of fibronectin and collagen IV (Figure 1(E)).

For the *in vivo s*tudy, the increase of BUN and Scr levels was found in STZ treated mice (Figure 1(F)), suggesting that the mouse model of DN was successfully established, which was further manifested by serious tubular injuries (H&E staining) and interstitial fibrosis (Masson staining) (Figure 1(G,H)). By qRT-PCR, we also confirm that TUG1 and DUSP6 in DN mouse kidney tissues were also decreased compared with normal control (Figure 1(I)). All the results demonstrated that TUG1 was down-regulated by hyperglycemia both *in vitro* and *in vivo*.

### 3.2. TUG1 overexpression slows down the DN progression by reducing inflammation and fibrosis

**Figure 2.**
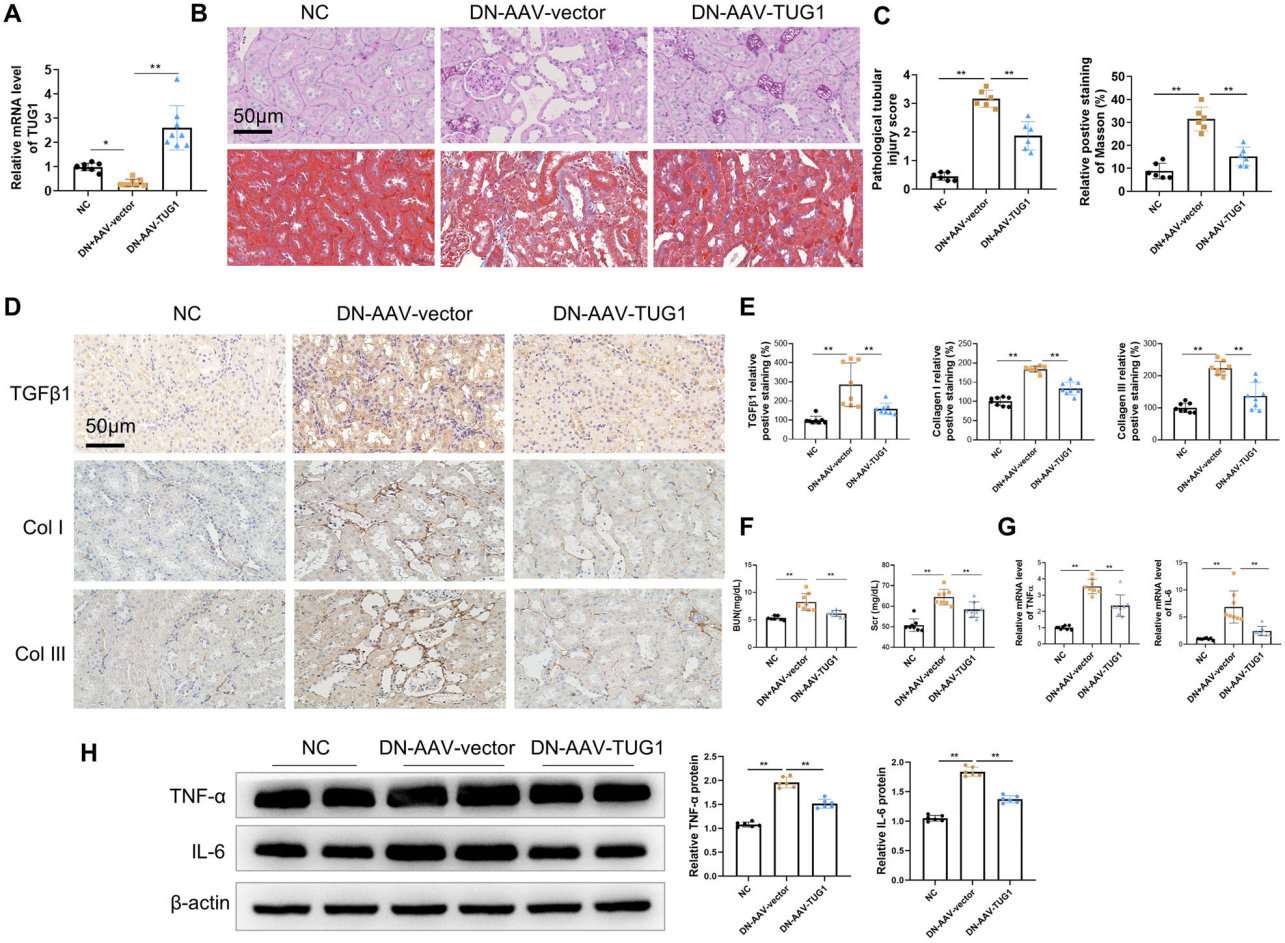
Delivery of AAV-TUG1 ameliorates kidney injuries in DN mice. (A) TUG1 expression increased significantly in kidney tissues in mice by AAV-mediated gene delivery; (B–C) AAV-TUG1 overexpression significantly attenuates the pathological injuries (HE staining analysis) and fibrosis (Masson’s trichrome) in DN mice. (D–E) AAV-TUG1 overexpression significantly attenuates the renal TGF-β1, collagen I and III protein level in DN mice by immunohistochemistry. (F) AAV-TUG1 overexpression significantly improves kidney function by decreasing BUN and Scr concentration; (G) AAV-TUG1 overexpression attenuates mRNA levels of TNFα and IL-6 in DN mice; (H) AAV-TUG1 overexpression attenuates protein levels of TNFα and IL-6 in DN mice.

To further investigate the effects of TUG1 on interstitial fibrosis of DN mice, we used AAVs to enhance Tug1 expression in DN mice. According to RT-qPCR analysis, TUG1 expression was significantly upregulated in the kidneys of mice by AAV-TUG1 treatment (Figure 2(A)). H&E and Masson’s trichrome staining results illustrated that overexpression of TUG1 attenuated the DN-induced tubular vacuolar degeneration of the mice, as well as interstitial fibrosis which was manifested by Masson staining (Figure 2(B,C)). Immunohistochemistry also demonstrated that TUG1 exogenous expression reduced the TGFβ1 expression in renal interstitium, and decreased the deposition of collagens type I & III in interstitium (Figure 2(D,E)). In addition, after the overexpression of TUG1, the serum levels of BUN and Scr were remarkably decreased in DN mice compared with normal group (Figure 2(F)). Moreover, TUG1 overexpression repressed the DN-induced increase in mRNA level and protein levels of several serum inflammatory cytokines, including TNFα and IL-6 (Figure 2(G,H)).

### 3.3. TUG1 inhibits inflammation and ECM secretion of HK-2 cell under high glucose stimulation

**Figure 3.**
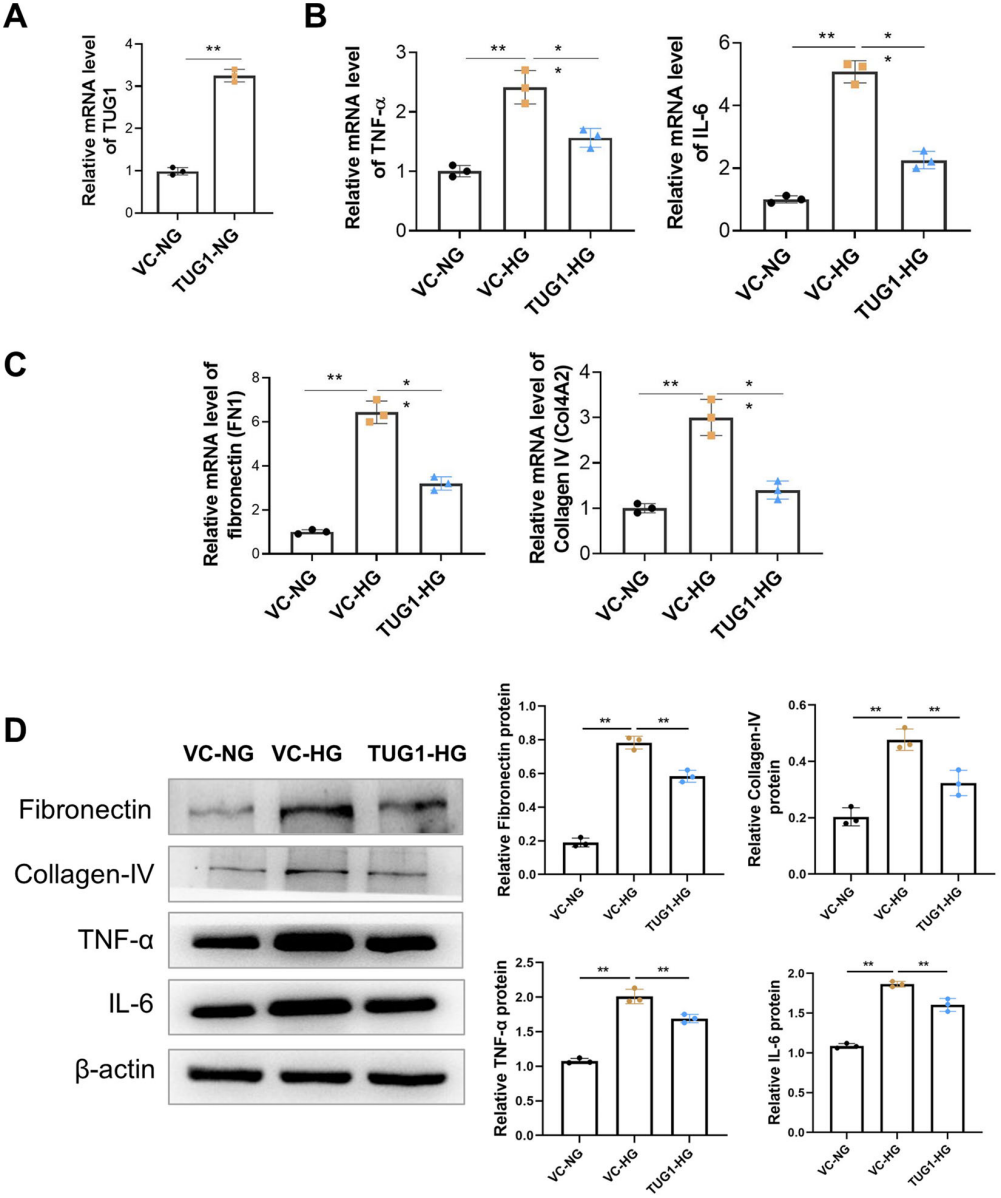
Over-expression of TUG1 reduces proinflammation and profibrotic caused by high glucose toxicity in HK2 cells. (A) Relative expression of TUG1 increased after TUG1 transfection; (B) mRNA levels of TNF-α and IL-6 in HG-treated HK2 cells were significantly reduced by overexpression of TUG1; (C) mRNA levels of fibronectin (FN1) and collagen IV (COL4A2) in HG-treated HK2 cells were significantly reduced by overexpression of TUG1; (D) protein levels of fibronectin, collgen IV, TNF-α and IL-6 by Western blot. **p < 0.01.

We explored the biological role of TUG1 in HG-stimulated HK-2 cells. Firstly, the stable upregulation of TUG1 was achieved by stable transfection of pcDNA3.1-TUG1 into HK-2 cells (Figure 3(A)). Further, we treated HK-2 cell with high glucose, as presented in Figure 3(B), by qRT-PCR, we found that HG stimulation caused significantly increased mRNA levels of TNF-α and IL-6 in the vector control (VC) group, but TUG1 overexpression significantly decreased mRNA expression of these cytokines. Meanwhile, HG stimulation increased the mRNA levels of fibronectin (FN1) and collagen IV (Col4A2) in HK2 cells, but these up-regulation were reversed by exogenous TUG1 over-expression (Figure 3(C)). The mRNA trends in each groups were consistent with protein levels tested by western blot (Figure 3(D)). All these results suggested that over-expression of TUG1 could down-regulated HG stimulated inflammation and fibronectin production in HK-2 cell.

### 3.4. miR-145-5p is a key downstream gene of TUG1

**Figure 4.**
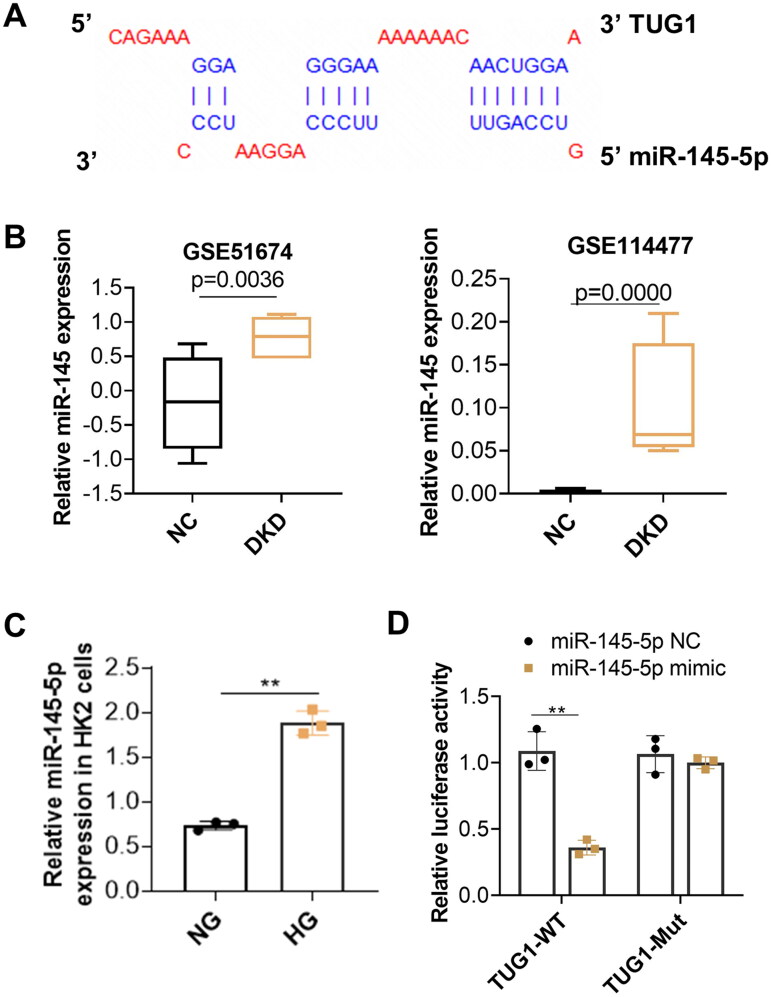
Inhibition of miR-145-5p alleviates tubular fibrosis under HG condition. (A) miR-145-5p is the target gene of TUG1. The potential binding sites between miR-145-5p and TUG1 were analyzed by online target tools; (B) MiR-145-5p expression is up-regulated in DN patients by GSE dataset GSE51647 and GSE 114477. (C) MiR-145-5p expression in HK2 cells increased after HG stimulation; (D) the binding between miR-145-5p to TUG1 was verified by the luciferase reporter assay. **p < 0.01.

To further explore the potential mechanism of TUG1 in HG-stimulated tubular impairment, RAID v2.0 (https://www.rna-society.org/raid2/index.html) was used to identify potential microRNA that interacts with TUG1. The potential micro RNAs with a confidence score above 0.5 were screened, and MiR-145-5p was selected for further analysis. The target prediction information was shown in Figure 4(A). The expressions of miR-145 in diabetic kidney disease (DKD) was analyzed subsequently in two GEO dataset (GSE51674 and GSE114477). Results showed expressions of miR-145 are upregulated in tubular tissues of diabetic patients (Figure 4(B)). Our *in vitro* analysis also demonstrated expression of miR-145-5p increased in high glucose treated HK2 cells (Figure 4(C)). We further conducted a dual luciferase report assay and revealed that miR-145-5p mimics notably decreased the luciferase activity of TUG1-WT but had no significant influence on that of the TUG1-Mut vector (Figure 4(D)). All these results implied that miR-145-5p was the downstream effector of TUG1.

### 3.5. Inhibition of miR-145-5p increases TUG1 expression and decreases expressions of collagen IV and fibronectin induced by HG

**Figure 5.**
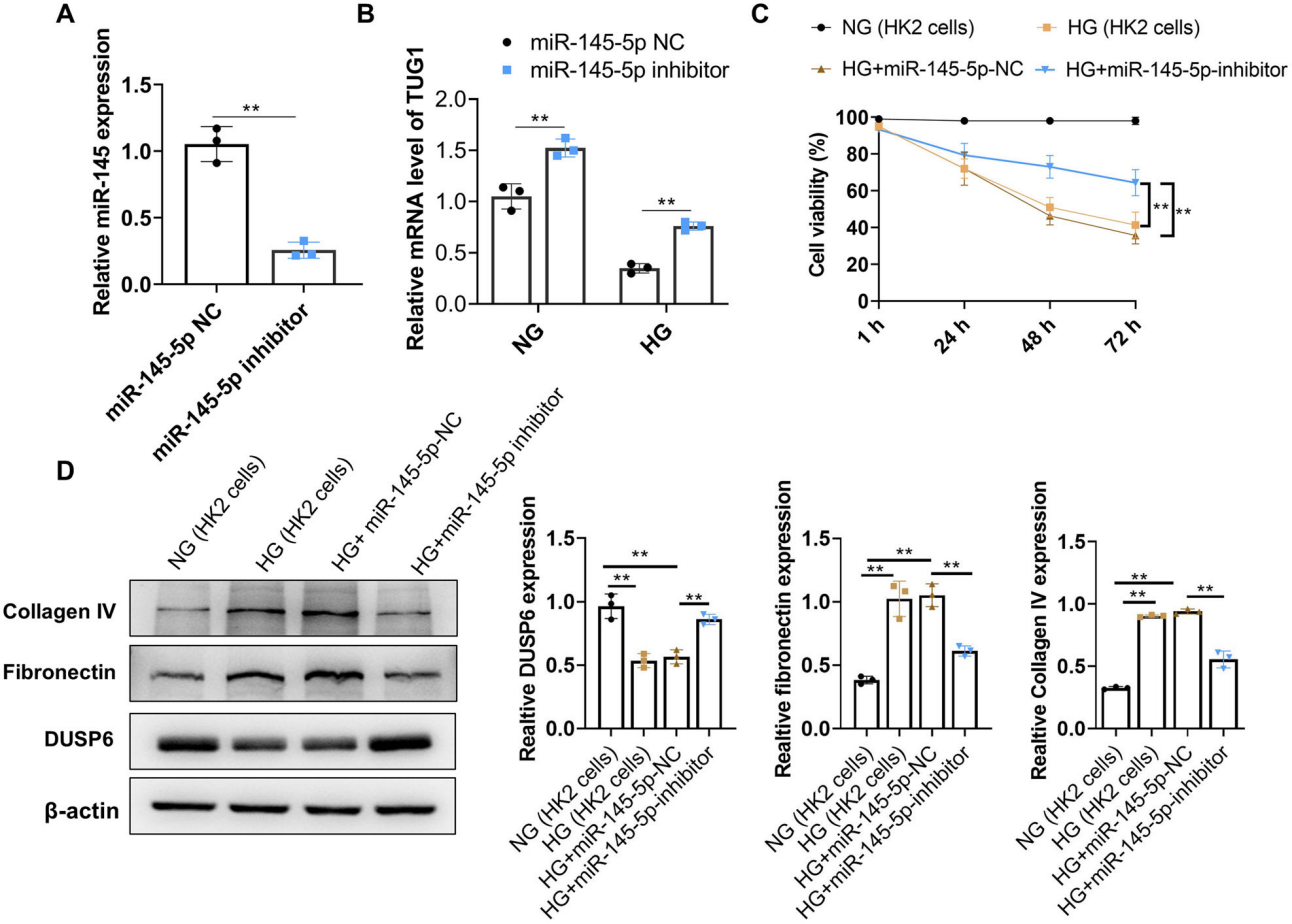
Inhibition of miR-145-55 significantly reduces the fibrosis characteristics in HG-treated HK2 cells. (A) Expression of miR-145-5p was significantly reduced by its inhibitor transfection. (B) TUG1 expression increased in NG and HG culture after miR-145-5p was inhibited; (C) cell viability of HK2 of miR-145-5p NC and miR-145-5p inhibitor under HG condition; (D) protein expressions of DUSP6, Collagen IV and fibronectin in miR-145-5p NC and miR-145-5p inhibitor group under HG condition.

We next evaluated the effect of downregulation of miR-145-5p on HK2 cells. The inhibitor of miR-145-5p was designed and transfected into HK2 cells. The transfecting efficacy was evaluated by qRT-PCR at 24h, 48 and 72h respectively after transfection, which demonstrated that miR-145-5p was significantly reduced by miR-145-5p inhibitor until 72h (Figure 5(A), only data at 72h was shown). Next step, transfected cells were incubated with normal glucose and high glucose for another 72h. Results showed that miR-145-5p inhibitor significantly increased the expression of TUG1 in HK2 cells both in NG and HG medium (Figure 5(B)). Furthermore, HG reduced the viability of HK2 cells but transfecting miR-145-5p inhibitor remarkably reverse this phenomenon (Figure 5(C)). Protein expressions of DUSP6, Collagen IV, and fibronectin in each group were analyzed by western blot. Results showed that inhibiting miR-145-5p increased protein expression of DUSP6, while decreased protein expression of collagen IV and fibronectin significantly (Figure 5(D)).

### 3.6. DUSP6 is the direct target gene of miR-145-5p

**Figure 6.**
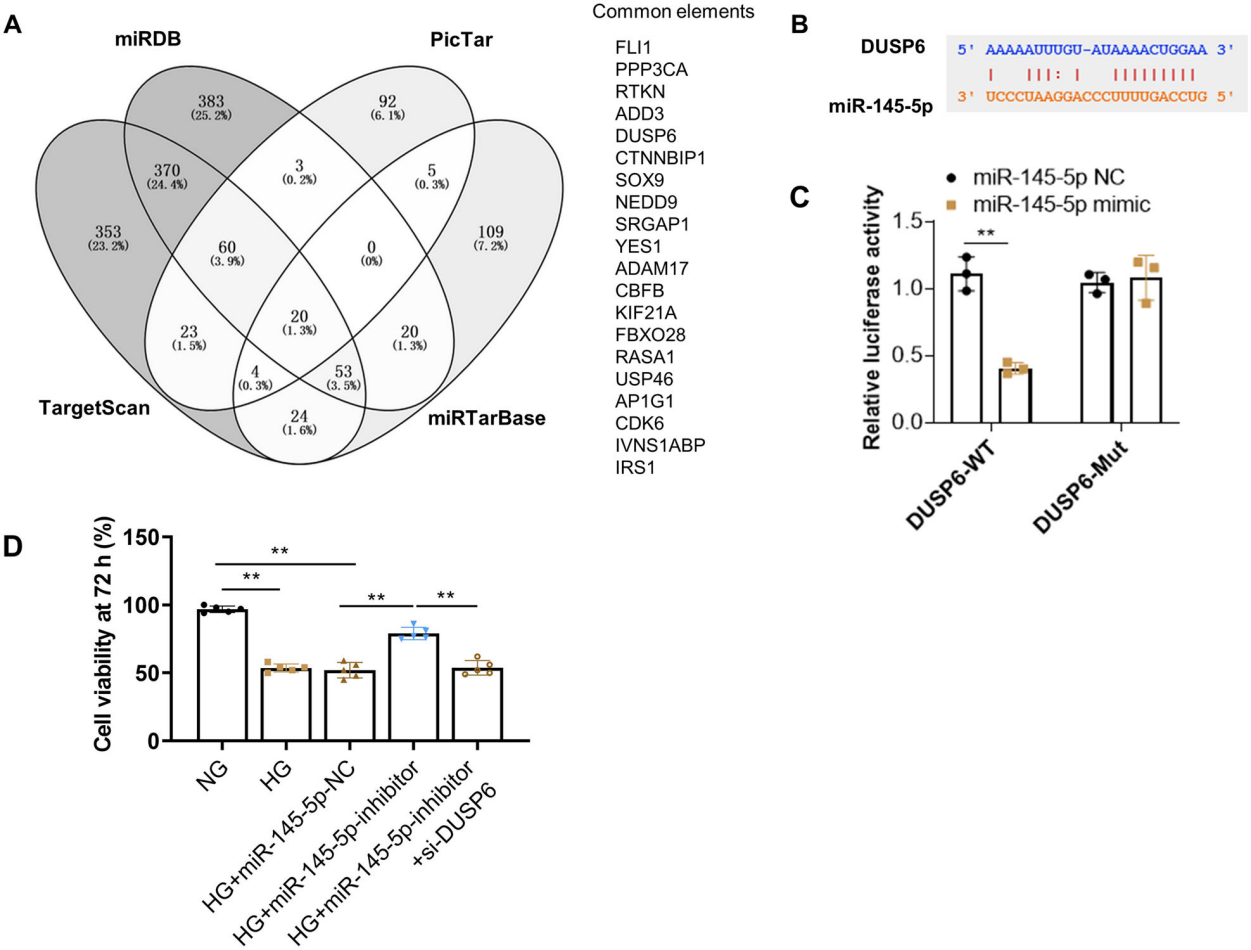
DUSP6 is the target gene of miR-145-5p. (A) potential targets of miR-145-5p were analyzed by online target tools; B the possible binding site of miR-145-5p to DUSP6 by bioinformatics prediction (C) Interaction between DUSP6 and miR-145-5p is verified by the luciferase reporter assay; (D) HK2 cell viability increased by miR-145-5p inhibition under HG condition, but was effectively abolished by DUSP6 knocking down. **p < 0.01.

Aiming to investigate whether DUSP6 was directly regulated by miR-145-5p, a further analysis of possible target genes of miR-145-5p was conducted using online predicting tools (TargetScan, miRDB, PicTar, and ENCORI). Results demonstrate a total of 20 genes could be a potential target of miR-145-5p, including DUSP6 (Figure 6(A)). TargetScan software was applied to identify the potential binding site of the DUSP6 gene for miR-145-5p, which was shown in Figure 6(B). Then the luciferase construct containing the wide type (WT) or mutation (Mut) binding sequence of DUSP6 was co-transfected with the miR-145-5p mimic or miR-NC into cells. The results demonstrated that the luciferase activity of the DUSP6-WT sequence was suppressed by the miR-145- 5p mimic but not the DUSP6-Mut sequence (Figure 6(C)), which showed that DUSP6 was the direct target of miR-145- 5p in HK2 cells. In addition, we found that increased HK2 cell viability caused by inhibition of miR-145-5p could be reversed by knocking down DUSP6 (Figure 6(D)).

### 3.7. TUG1 alleviates renal tubular fibrosis in HK2 cells in DUSP6 dependent manner in DN mice

**Figure 7.**
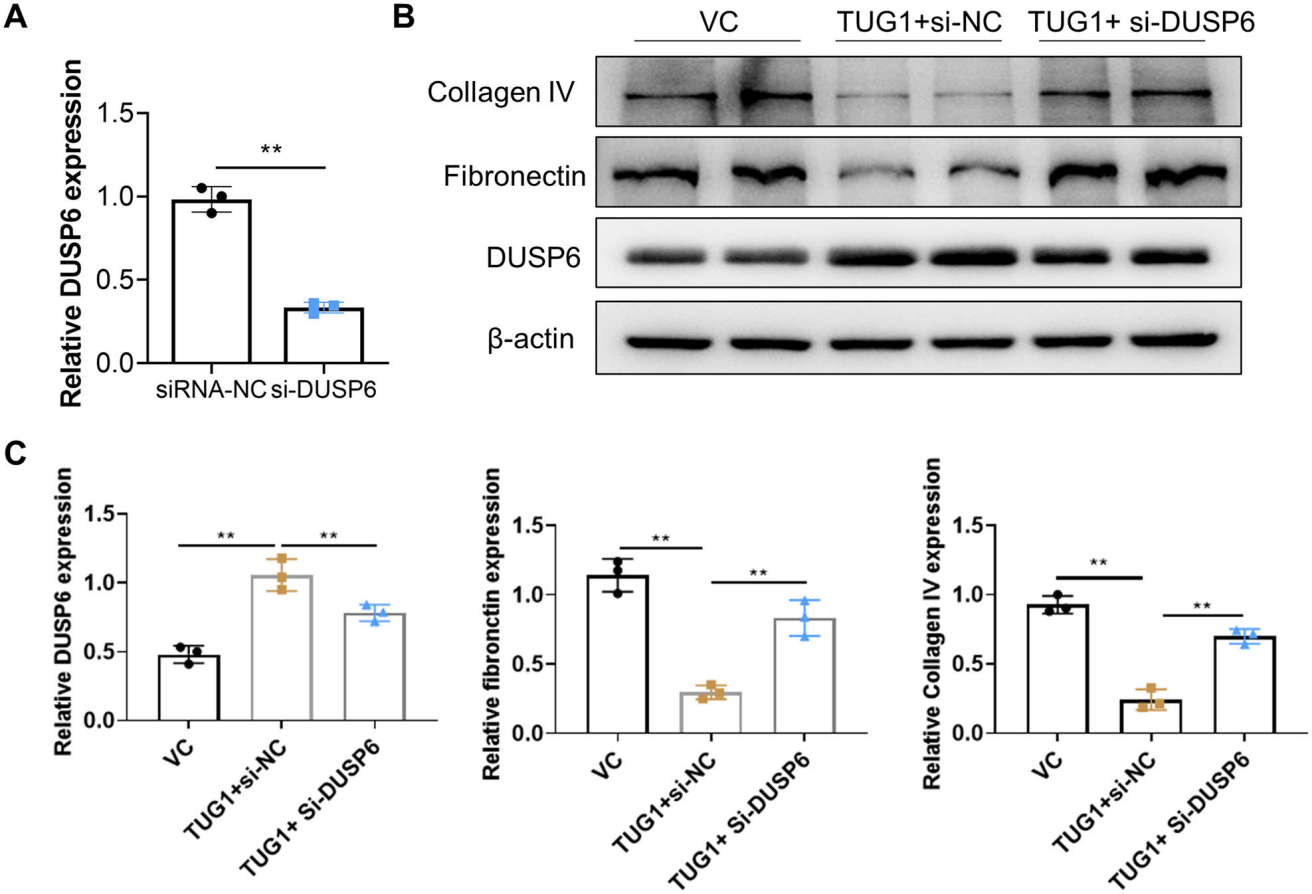
Knocking down DUSP6 abolishes the TUG1 effect on reducing fibrosis in HG-treated HK2 cells. (A) DUSP6 was knocked down in TUG1 stable expressed HK2 cells; (B–C) Reducing collagen IV and fibronectin ability of TUG1 was abolished by silencing DUSP6 in HG-treated HK2 cells confirmed by western blot and quantitative analysis. **p < 0.01.

To confirm whether TUG1 plays its anti-fibrosis effect via DUSP6, the DUSP6 gene expression was silenced by transient siRNA-DUSP6 transfection in stable over-expressing TUG1 HK2 cells. The knocking down efficacy of DUSP6 in TUG1 over-expression HK2 cells was confirmed by RT-qPCR, and mRNA expression of DUSP6 was significantly decreased by transfection of si-DUSP6 (Figure 7(A)). When these cells were cultured under HG condition, as presented in Figure 7(B), we found out that the anti-fibrosis of upregulation of TUG1 in HG condition was significantly abolished by silencing DUSP6, manifested by reversed production of fibronectin and collagen IV. All these results confirmed that protective effect of TUG1 was dependent with DUSP6.

## 4. Discussions

Renal tubular injury occurred during the progression of diabetes, and tubular impairment (including vacuolar degeneration and interstitial fibrosis) is dominant in DN pathological injuries [25]. The occurrence of fibrosis in renal tubular epithelial cells is mainly affected by local transforming growth factor- β (TGF- β), epidermal growth factor (EGF), connective tissue growth factor (CTGF), and platelet-derived growth factor (PDGF), and other cytokines [26]. TGF-β is recognized as an important cytokine to induce fibrosis. HK2 is a normal human proximal convoluted tubular epithelial cell, which has the phenotypic markers and physiological and biochemical characteristics of human renal tubular epithelial cells. Therefore, HK2 was selected as the cell model in the current study. From our analysis, high glucose toxicity leads to higher expression of fibronectin and collagen IV, which suggested that HG can induce the fibrosis in HK2 cells. Meanwhile, downregulation of TUG1 and DUSP6 expression was also observed in HG-treated HK2 cells and DN kidneys.

Long non-coding RNA (LncRNA) has almost no protein-coding ability but has a wide range of biological functions. As an evolutionarily conserved Inc RNA, TUG1 could regulate mitochondrial bioenergy in DN [27]. The study also found that the expression of TUG1 in the podocytes of diabetic mice decreased significantly while upregulating TUG1 could improve mitochondrial function and improve DN podocyte injury. TUG1 is also a regulator of lung fibrosis caused by hypoxia, and an important regulator of cardiac fibroblast-myofibroblast transformation [28]. In the current study, by *in vitro* study, we confirmed that over expression of TUG1 could significantly reduce the Collagen IV and fibronectin expression in HG-treated HK2 cells; Meanwhile, by AAV-TUG1 lentivirus vector delivery, we also confirm that exogenous TUG1 administration also significantly improve the renal function and ameliorate the interstitial fibrosis in DN mice. All these results prove that TUG1 has satisfactory renal protective effect.

Aims to clarify the mechanisms of TUG1 in renal protection, we use the informatics methods to screen its possible downstream molecules, and finally, TUG1-miR-145-5p-DUSP6 axis emerged, and further identified by luciferase assay. Rescue assay also demonstrated that TUG1 plays its renal beneficial effect is dependent on this axis. At beginning, we explored the potential micro RNAs via RAID v2.0 and finally selected MiR-145-5p for further analysis. In fact, there are several other possible targets, but based on the novelty and binding capabilities, the Mir-145-5p has a higher binding ability. There are total 15 nucleotides in miR-145-5p can bind to TUG1. I also tested the other micro RNAs and their downstream proteins, but not effective as Mir-145-5p/DUSP6 in high glucose induced tubular fibrosis.

Collagen IV and fibronectin proteins are the main components of the extracellular matrix (ECM). The abnormal increase of collagen IV in the glomerulus could cause glomerular disease and the increase of fibronectin protein suggests the existence of renal fibrosis [29]. In addition, the increase in collagen IV and fibronectin synthesis will accelerate the progress of diabetic nephropathy (DN) [30]. In addition to the classic TGF- β/Smads signaling pathway, there are several various of cellular signal pathways to regulate the occurrence and development of fibrosis, such as MAPK and Wnt/β - Catenin, mTOR pathway, etc. [13]. The activation of the ERK1/2 MAPK signal pathway has been reported to be involved in TGF-β induced kidney fibrosis, through direct or indirect processes, TGF-β activates ERK1/2, which further phosphorylates downstream target proteins and promotes the process of cell fibrosis [14].

Dual-specificity phosphatases (DUSPs) are negative regulators of MAPK signal transduction and can dephosphorylate p38, JNK, and ERK under different conditions [15]. Extracellular signal-regulated kinases 1 and 2 (ERK1/2) are important extracellular signals transductor. DUSP6 can specifically dephosphorylate ERK1/2 on tyrosine and serine/threonine residues, resulting in the inactivation of ERK1/2 *in vivo* and *in vitro*. Consistent with a previous report [17], we demonstrated upregulation of DUSP6 could alleviate fibrosis of the kidney. Pfuhlmann et al. [31] indicated that DUSP6 deficiency is associated with impaired systemic glucose tolerance. Our study showed that the expression of DUSP 6 was downregulated in the kidney under the stimulation of glucose toxicity, and this low expression is caused by the upregulation of miR-145-5p. Some research shows that downregulating miR-145-5p can reduce the inflammatory response and diabetic retinopathy progress [32]. Shahrokhi et al. [33] analyzed plasma miR-145-5p expressions in healthy individuals, prediabetic patients, and diabetic patients, and they indicated that miR-145-5p was down-regulated in the prediabetics and T2D patients compared to the controls. While Zamanian Azodi et al. [34] reported expressions of miR-145-5p were upregulated significantly in patients with gestational diabetes mellitus in 2 GSE datasets. Is the difference in expression due to the specificity of organ tissues or the different stages of diabetes? The mechanism of diabetes and its complications is complex and is influenced by the environment and genes, these all need further analysis in follow-up research.

There were several limitations for the current research. Firstly, we don’t know exactly the upstream and downstream relationships between miR-145-5p and TUG1, and secondly, we did not investigate the effect of TUG1/miR145-5p/DUSP6 axis in glomerular compartment.

In a conclusion, our research provides some meaningful information: high glucose toxicity resulted in down-regulation of lncTUG1 expression in renal tubules, further up-regulating the expression of miR-145-5p, then downregulating the expression of anti-fibrosis factor DUSP6, the target of miR-145-5p. LncTUG1-miR-145-5p-DUSP6 axis plays an important role in DN, especially in tubular fibrosis.

## Ethical approval

All animal experiment was reviewed and approved by the Animal Care Committee of Affiliated Hospital of Hebei University of Engineering. All study was performed according to the international, national, and institutional rules considering animal experiments.

## Supplementary Material

Supplemental Material

## Data Availability

The data of the current study are available from the corresponding author on reasonable request.
