# Supplementary figures and images for: LncTUG1 ameliorates renal tubular fibrosis in experimental diabetic nephropathy through the miR-145-5p/dual-specificity phosphatase 6 axis
Source: Ren Fail. 2023 Feb 16;45(1):2173950. doi: 10.1080/0886022X.2023.2173950 (PMC9937007; doi:10.1080/0886022X.2023.2173950)

**Figure 1**

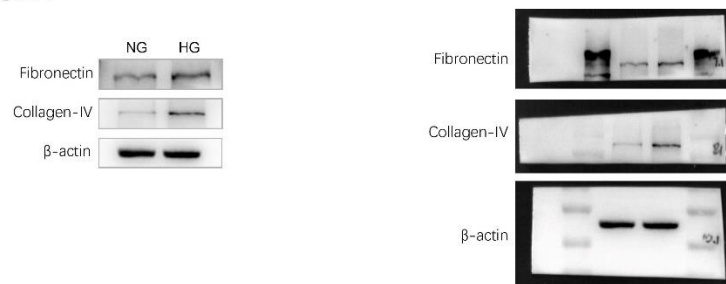

**Figure 2**

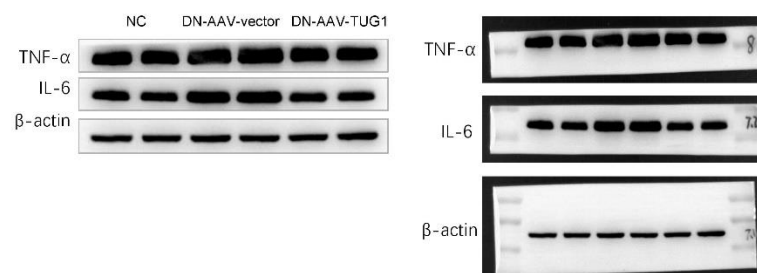

**Figure 3**

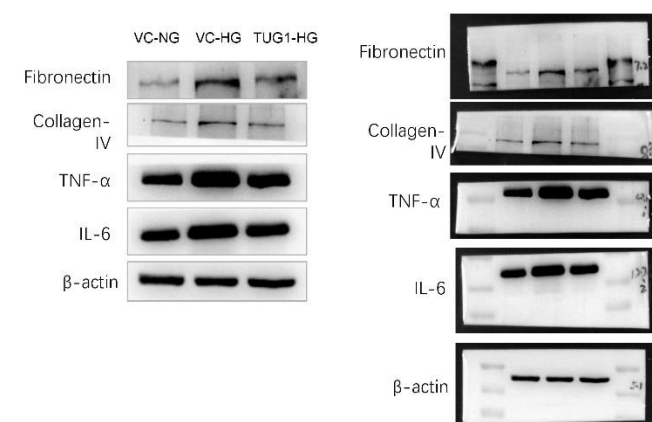

**Figure 5**

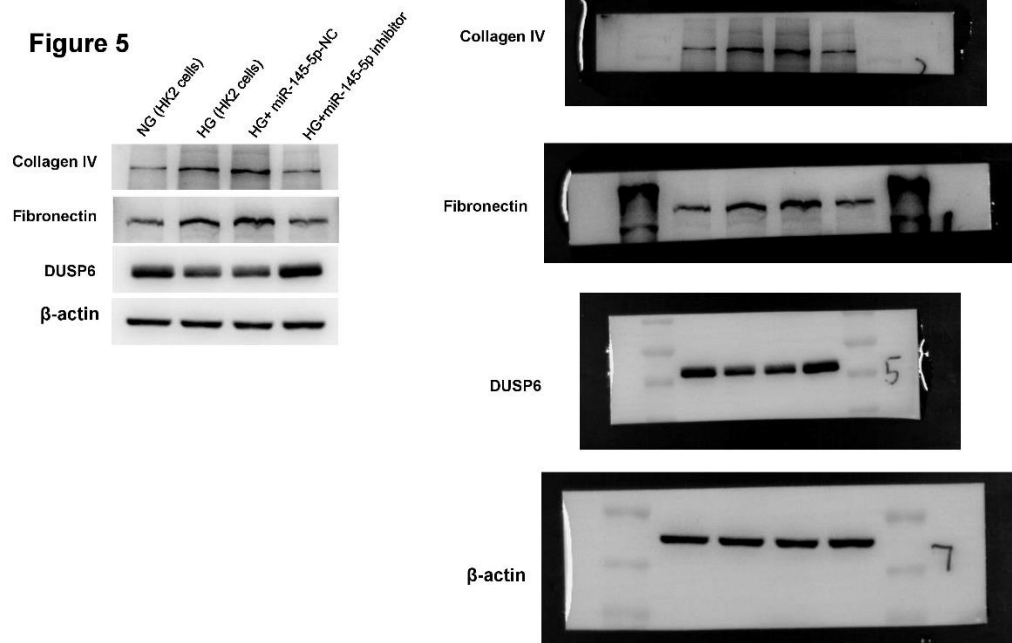

**Figure 7**

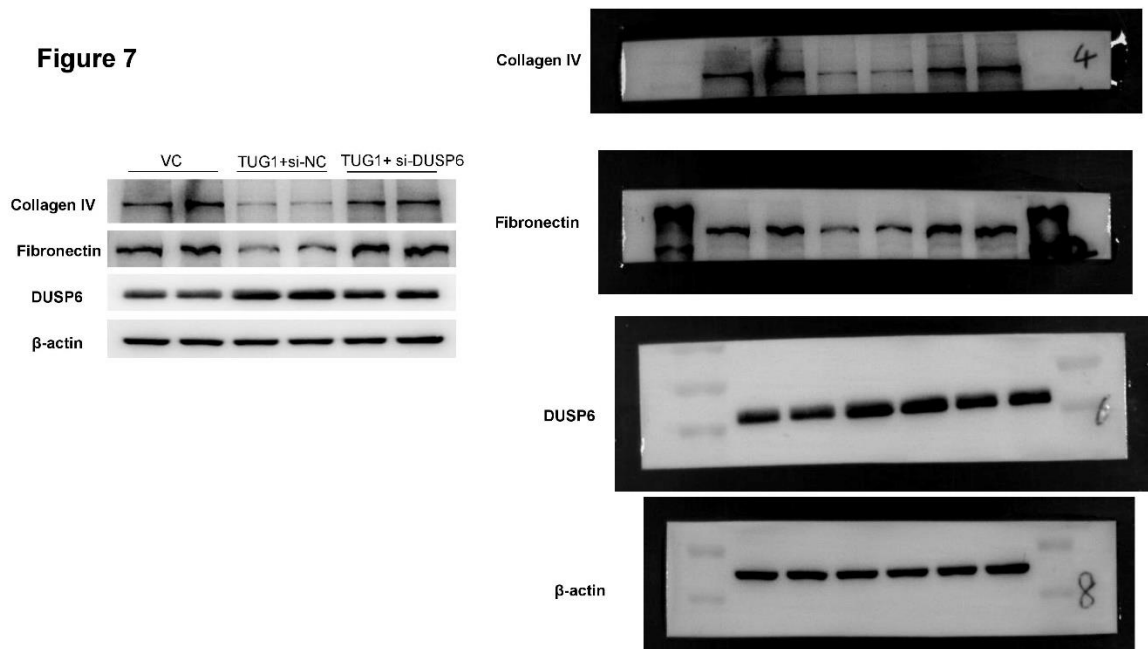

Supplement: Supplemental Material [file IRNF_A_2173950_SM9956.pdf]
